# Protection against Ovariectomy-Induced Bone Loss by Tranilast

**DOI:** 10.1371/journal.pone.0095585

**Published:** 2014-04-21

**Authors:** Tien Van Phan, Ke Ke, Ok-Joo Sul, Yun-Kyung Park, Kack-Kyun Kim, Yeon-Soo Cho, Hun-Taeg Chung, Hye-Seon Choi

**Affiliations:** 1 Department of Biological Sciences, University of Ulsan, Ulsan, Korea; 2 Department of Dentistry, Seoul National University, Seoul, Korea; INSERM U1059/LBTO, Université Jean Monnet, France

## Abstract

**Background:**

Tranilast (N-(3′,4′-dimethoxycinnamonyl) anthranilic acid) has been shown to be therapeutically effective, exerting anti-inflammatory and anti-oxidative effects via acting on macrophage. We hypothesized that Tranilast may protect against oxidative stress-induced bone loss via action in osteoclasts (OCs) that shares precursors with macrophage.

**Methodology and Principal Findings:**

To elucidate the role of Tranilast, ovariectomy (OVX)-induced bone loss in vivo and OC differentiation in vitro were evaluated by µCT and tartrate-resistant acid phosphatase staining, respectively. Oral administration of Tranilast protected against OVX-induced bone loss with decreased serum level of reactive oxygen species (ROS) in mice. Tranilast inhibited OC formation in vitro. Decreased osteoclastogenesis by Tranilast was due to a defect of receptor activator of nuclear factor-κB ligand (RANKL) signaling, at least partly via decreased activation of nuclear factor-κB and reduced induction and nuclear translocation of nuclear factor of activated T cells, cytoplasmic 1 (or NFAT2). Tranilast also decreased RANKL-induced a long lasting ROS level as well as TGF-β to inhibit osteoclastogenesis. Reduced ROS caused by Tranilast was due to the induction of ROS scavenging enzymes (peroxiredoxin 1, heme oxygenase-1, and glutathione peroxidase 1) as well as impaired ROS generation.

**Conclusions/Significance:**

Our data suggests the therapeutic potential of Tranilast for amelioration of bone loss and oxidative stress due to loss of ovarian function.

## Introduction

Postmenopuasal osteoporosis has been attributed to loss of ovarian function, which leads to bone loss through increased bone resorption over bone formation. The increase in bone resortiopn is due to the increases of differentiation and/or survival of osteoclast (OC) as well as its activity [1], [2]. OCs are multinucleated giant cells responsible for bone resorption, differentiate from hematopoietic cells of monocyte/macrophage lineage, and share some morphological and functional properties with macrophages. They are responsible for not only physiological bone remodeling, but also for bone destruction associated with chronic inflammatory disease [3]. During osteoclastogenesis two molecules which are generated from bone marrow mesenchymal cells are essential: macrophage-colony stimulating factor (M-CSF) and receptor activator of nuclear factor-κB ligand (RANKL), a member of the tumor necrosis factor (TNF) family [4]. RANKL which is both necessary and sufficient for osteoclastogenesis in the presence of M-CSF, enhances OC activity, and prolongs OC survival by decreasing apoptosis [5]. Stimulation of the receptor, RANK by binding RANKL activates the key transcription factors, nuclear factor-κB and nuclear factor of activated T cells, cytoplasmic 1 (NFAT2), resulting in expression of OC-specific genes such as tartrate-resistant acid phosphatase (TRAP) and cathepsin K [6], [7], [8]. Ectopic expression of NFAT2 results in undergoing osteoclastogenesis in the absence of RANKL and embryonic stem cells devoid of NFAT2 fail to differentiate into OC [6], implying that NFAT2 plays a critical role in osteoclastogenesis.

Tranilast, N-(3′,4′-dimethoxycinnamonyl) anthranilic acid, has been developed as an anti-allergic drug, by inhibiting the release of chemical mediators from mast cells and basophils [9]. The drug shares the anthranilic acid core with endogenous 3-hydroxyanthranilic acid, a tryptophan metabolite in indoleamine 2,3-dioxygenase pathway [10]. Additionally the drug has been shown to exhibit anti-inflammatory effects via inducing hemoxygenase-1 (HO-1) and suppressing release of proinflammatory cytokines [11], and inhibit tumor growth [12]. Tranilast has been demonstrated to suppress prostate cancer cell proliferation along with reduced cranial bone defects [12]. However, the relevant action mechanisms of Tranilast have not been clearly elucidated yet.

We hypothesized that Tranilast may protect against bone loss upon oxidative stress via action in OCs. In the present study, we demonstrated the protective role of Tranilast against OVX-induced bone loss, exerting a decreased osteoclastogenesis.

## Results

### Tranilast Protects against OVX-induced Bone Loss in vivo

**Figure 1:**
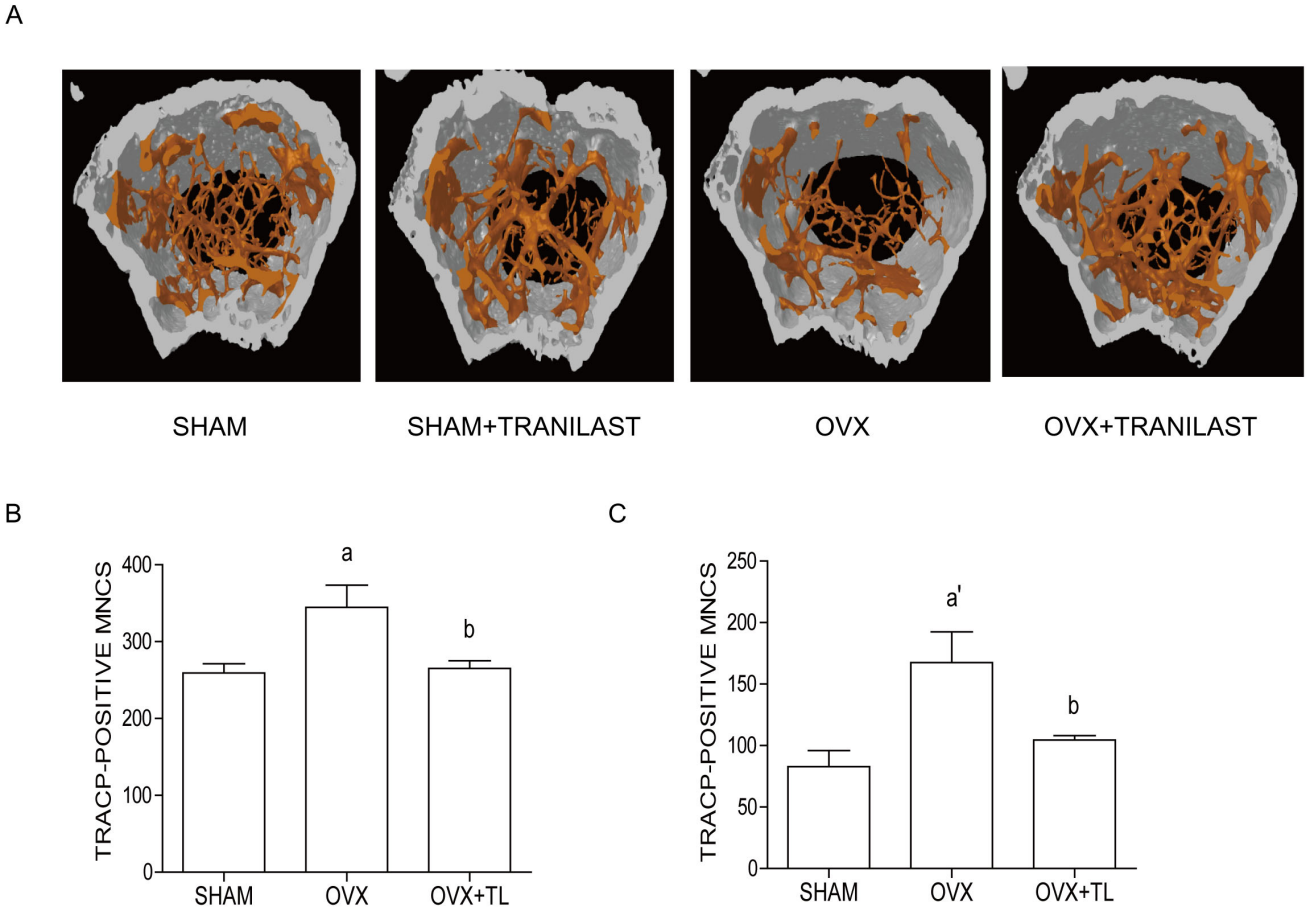
Tranilast attenuates OVX-induced bone loss in mice. Bone densities of the femora were measured from vehicle-treated (sham, n = 6; OVX, n = 6), Tranilast (200 mg/kg/d)-treated (sham, n = 6; OVX, n = 6) mice 8 weeks after surgery. The representative µCT images of distal femora (1.0 mm from the growth plate of the distal femur) (A). Number of OCs in the cultures of enriched BMM (B) and whole bone marrow (C) stimulated with RANKL/M-CSF and 1,25(OH)_2_D_3_ (C), respectively. a, *p*<0.05; a’, *p*<0.01 compared with vehicle-treated SHAM. b, *p*<0.05 compared with vehicle-treated OVX. No significant difference between vehicle-treated SHAM and Tranilast-treated OVX (B, C).

To examine the effect of Tranilast on OVX-induced bone loss, we analyzed µCT of femurs from OVX mice treated with Tranilast or vehicle, and compared the effects with those of sham surgery. At 14 week of age, no significant differences in body size and shape were observed between Tranilast and vehicle-treated OVX mice. Administration of Tranilast protected against OVX-induced bone loss, but had no significant effect on sham mice (**Fig. 1** **,** **Table 1**). Tranilast induced significant increases of attenuated bone mineral density (BMD), bone volume (BV/TV), and trabecular number (Tb. N.) and a decrease of enlarged trabecular space (Tb. Sp.) after OVX (**Table 1**). Consistent with these findings, serum CTX-1, a marker of in vivo bone resorption was significantly reduced in the Tranilast-administered OVX mice, whereas TRACP5b, a representation of the number of OC was decreased without any statistical significance (**Table 1**). Ex vivo cultures of bone marrow-derived macrophage (BMM) enriched population from Tranilast-treated OVX mice showed a significant decrease compared with those from OVX mice (Fig. 1B), suggesting that in vivo administration of Tranilast induced decreased number of OC. However, in vivo bone formation marker, serum alkaline phosphatase (ALP) and osteocalcin were not significantly changed by the treatment of Tranilast (**Table 1**), suggesting that protective effect of Tranilast on bone may be due to action in OC. In agreement with this, ex vivo cultures of whole bone marrow exhibited a similar pattern as those of enriched BMM (Fig. 1C). Along with these bone parameters, serum H_2_O_2_ level was also significantly reduced by Tranilast (**Table 1**).

**Table 1:** Trabecular microarchitecture and biochemical markers of OVX and SHAM mice treated with vehicle or Tranilast at 8 week after surgery.

	SHAM		OVX	
	V	Tranilast	V	Tranilast
BMD [mg/cm^3^]	107.417±6.383	94.368±2.882	63.014±2.074^a”^	79.977±3.779^a”b^
BV/TV (%)	5.356±0.4449	4.463±0.2118	2.708±0.1665^a”^	3.936±0.3076^b^
Tb. N. [mm^−1^]	1.158±0.0652	0.997±0.0412	0.646±0.0313^a”^	0.861±0.0553^a’b^
Tb. Sp.(×10^3^) [mm]	290.000±6.200	297.700±6.700	377.900±7.900^a”^	352.000±6.500^a’’b^
Serum CTX-1 [mg/ml]	26.067±0.5691	24.590±0.7316	42.456±1.238^a”^	38.783±0.9799^a’’b^
Serum TRACP5b [U/L]	5.060±0.07483	4.800±0.1549	9.983±0.7705^a”^	7.588±0.6909^a^
Serum ALP [U/L]	52.780±7.804	49.840±5.416	94.780±7.432^a’^	93.280±8.058^a’^
Serum OCN [ng/ml]	24.577±2.927	25.039±1.923	35.133±2.630^a^	30.115±1.874
Serum H_2_O_2_ [nmol/ml]	57.346±1.428	59.594±1.290	64.509±0.674^a’^	59.291±1.376^b^

Data are represented as mean±SEM. Differences between each groups were analyzed by one-way ANOVA, followed by Bonferroni post tests. a, *p*<0.05; a’, *p*<0.01; a”, *p*<0.001 compared with vehicle-treated SHAM. b, *p*<0.05 compared with vehicle-treated OVX. No significantly difference between vehicle-treated SHAM vs. Tranilast-treated SHAM.

### Tranilast Inhibits OC Formation

**Figure 2:**
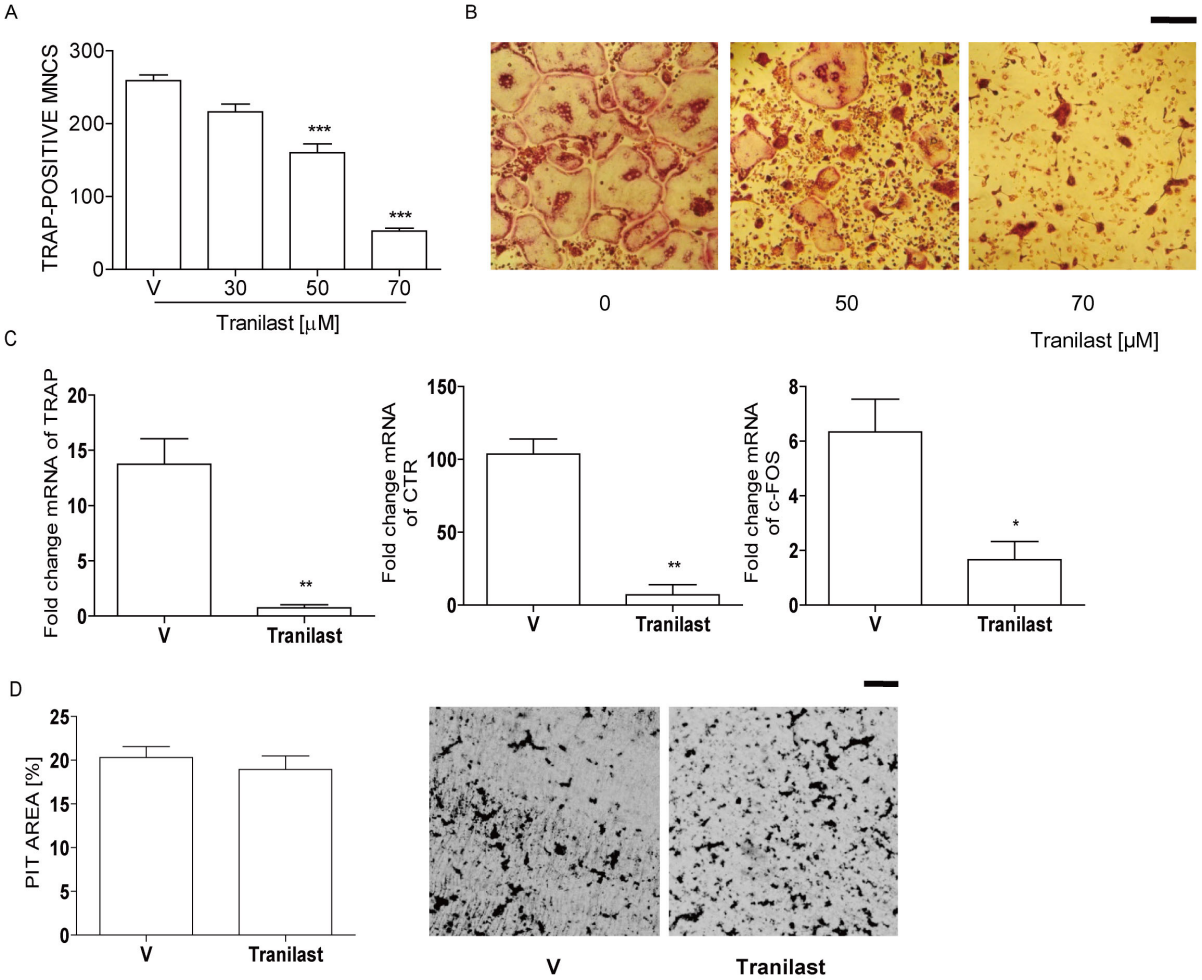
Tranilast decreases OC formation induced by RANKL. (A) BMM were incubated with Tranilast (30, 50, 70 µM) in the presence of M-CSF (20 ng/ml) and RANKL (40 ng/ml). After 3 d, cells were fixed and stained for TRAP. Means of 4 groups are significantly different (*P*<0.001). ***, *P*<0.001 compared with V (vehicle)-treated cells. (B) Representative photos of A. Scale bar, 200 µm. (C) BMMs were incubated with Tranilast (70 µM) in the presence of M-CSF and RANKL for 48 h, total RNA was extracted and subjected to qPCR analysis for TRAP, calcitonin receptor (CTR), and c-Fos. *, *P*<0.05; **, *P*<0.01 with V. (D) RANKL-induced mature OC (∼1000 cells) was incubated with or without Tranilast (70 µM) on dentine slices for 24 h, and stained for pit formation. Representative photos of the resorption pits in V- and Tranilast-treated slices are shown. Scale bar, 50 µm. There was no significant difference between them in the areas of the resorption pits as determined with the ImageJ 1.37v program. Similar results were obtained in three independent experiments.

To evaluate whether Tranilast affects osteoclastogenesis, we determined the effects of Tranilast on OC formation in cultures of BMM free of stromal cells and lymphocytes. In the presence of the two osteoclastic cytokines, M-CSF and RANKL, maximal OC formation occurred after 3 d. OC formation was decreased by Tranilast in a dose-dependent manner by counting TRAP-positive MNC (**Fig. 2A, B**). Consistent with this result, after 48 h of RANKL stimulation, transcripts of TRAP, calcitonin receptor, and c-Fos were significantly lower in Tranilast-treated cells compared with vehicle-treated cells (**Fig. 2C**). To assess whether the decreased OC formation is due to retarded cell growth by Tranilast, we examined the proliferation of the BMM upon stimulation with M-CSF. There was no significant difference in proliferation of BMM when Tranilast was added (data not shown). We also assessed whether the decreased number of OC by Tranilast is due to increased death of mature OC. Tranilast did not change significantly survival of mature OC (data not shown). Next, we assessed whether Tranilst affects bone resorption. Mature OC gave rise to form substantial amounts of pits on dentine slices, but no further changes were found by Tranilast (**Fig. 2D**), suggesting that Tranilast did not affect OC activity.

### Tranilast Attenuates RANKL Signaling by Affecting Two Key Transcription Factors, NF-κB and NFAT2

**Figure 3:**
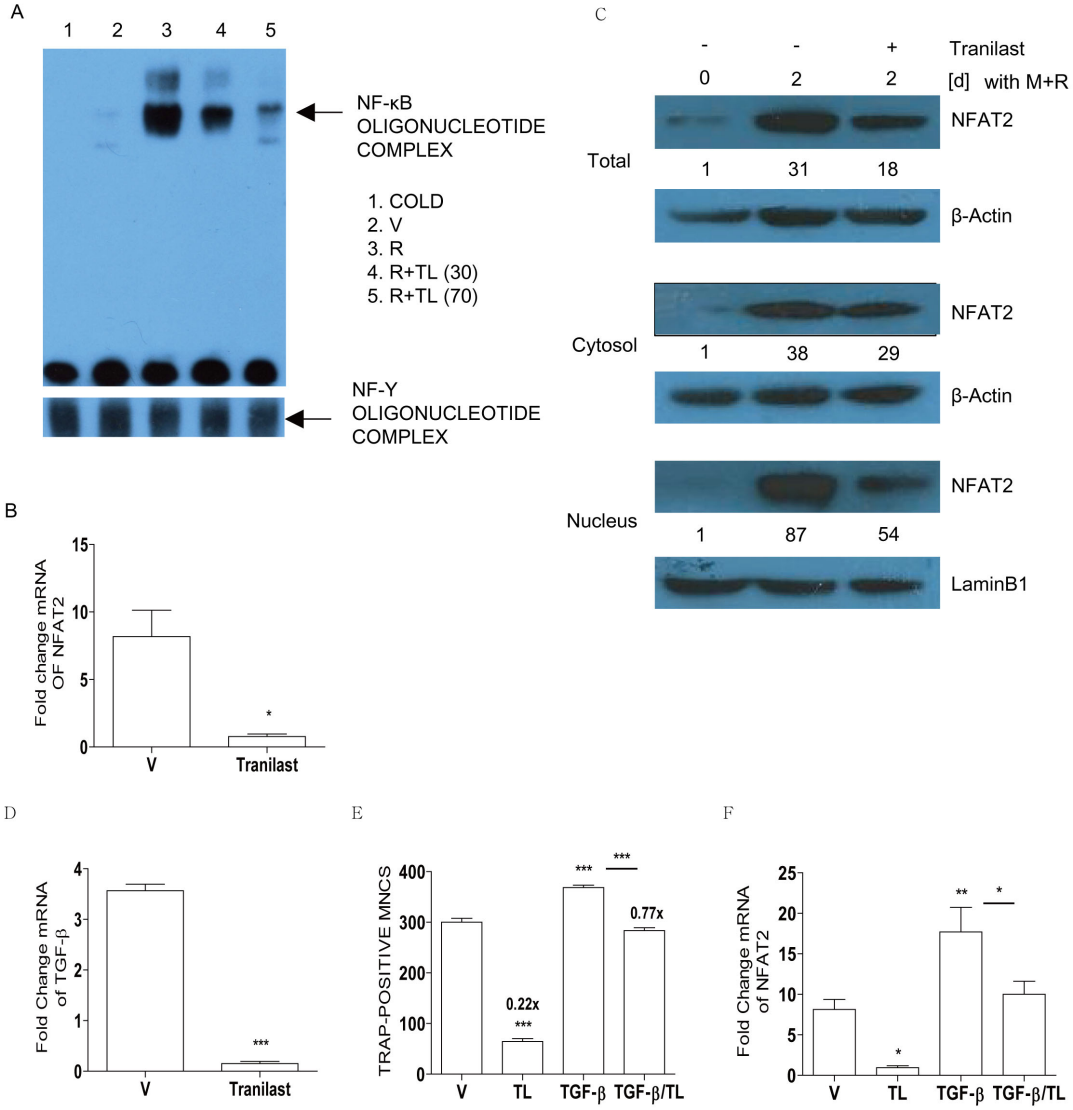
Tranilast impairs a RANKL signaling. (A) BMM (5×10^6^ cells/plate) was stimulated with vehicle (V) (lane 2) or RANKL (lane 3) with Tranilast (30 µM, lane 4; 70 µM, lane 5) for 1 h. Hundred-fold excess of unlabeled probe (lane 1) was used as a negative control. NF-Y DNA binding activity was measured as an internal control. (B–F) BMM with or without Tranilast (T, 70 µM) or TGF-β (10 ng/ml) were incubated with M-CSF and RANKL for 48 h (B–D, F) and 72 h for counting TRAP-positive MNCs (E). Total RNA was extracted and subjected to qPCR analysis for NFAT2 (B, F) or TGF-β (D). The expression level before RANKL treatment was set to be 1. * *P*<0.05, ** *P*<0.01, *** *P*<0.001 compared with V. Whole cell extracts, cytoplasmic fractions, and nuclear fractions were harvested from cultured cells and subjected to Western blot analysis with specific Abs as indicated. Abs for β-actin and lamin B1 were used for the normalization of cytoplasmic and nuclear extracts, respectively. Numbers between the panels are ratios of the intensity of NFAT2 over β-actin (total and cytosolic) or lamin B1 (nucleus) (C). ***, *P*<0.001 compared with V. Numbers above the histograms are ratios of the numbers of MNC formed in the presence of Tranilast to the numbers formed in its absence (E). There was a significant difference between TGF-β and TGF-β/TL (*** *P*<0.001; E, * *P*<0.05; F), whereas no significant difference between V and TGF-β/TL (E, F). Similar results were obtained in three independent experiments.

To gain molecular insights into the attenuating role of Tranilast during OC differentiation, we investigated the effect of Tranilast on RANKL-induced signaling pathways. First we assessed whether Tranilast affects RANKL-induced NF-κB activation by EMSA. RANKL stimulation of BMM induced NF-κB DNA binding activity (lane 3), and Tranilast decreased this activity in a dose-dependent manner (lane 4, 5) (**Fig. 3A**). The specificity of the binding activity was confirmed by competition assays using an excess unlabeled probe (lane 1).

Next, exposure of BMM to RANKL for 48 h increased the transcript of NFAT2, and the level of NFAT2 was significantly lower in the presence of Tranilast when compared with vehicle (**Fig. 3B**). We examined the expression and cellular location of NFAT2 protein in RANKL-stimulated BMM undergoing differentiation into multinucleated OCs. As shown in **Fig. 3C** (upper panel), a reduced level of total NFAT2 protein was observed in Tranilast-treated cells compared with vehicle-treated cells. RANKL stimulation induced enrichment of NFAT2 in the nucleus region of OCs, but it was lower in that of Tranilast-treated OCs. The reduction in the nucleus was greater than that in the cytosol (**Fig. 3C**, middle and bottom panels), suggesting that Tranilast reduced nuclear localization as well as total protein level of NFAT2 in OCs.

Then, we further investigated whether Tranilast inhibits OC formation via modulating the expression level of TGF-β, since Tranilast suppresses TGF-β in bone-derived stromal cells [13], and TGF-β induces NFAT2 in OC [14]. As shown in **Fig. 3D**, Tranilast resulted in dramatic decrease of RANKL-induced TGF-β in OC. Exogenous TGF-β increased RANKL-stimulated OC formation significantly and alleviated the inhibitory effect of Tranilast on osteoclastogenesis by counting TRAP-positive MNCs, but not completely (**Fig. 3E**). The decreased transcript of NFAT2 due to Tranilast was also restored partially by exogenous TGF-β (**Fig. 3F**), indicating alternative actions to explain the inhibitory effect of Tranilast on osteoclastogenesis.

### Tranilast Decreases RANKL-induced ROS Level

**Figure 4:**
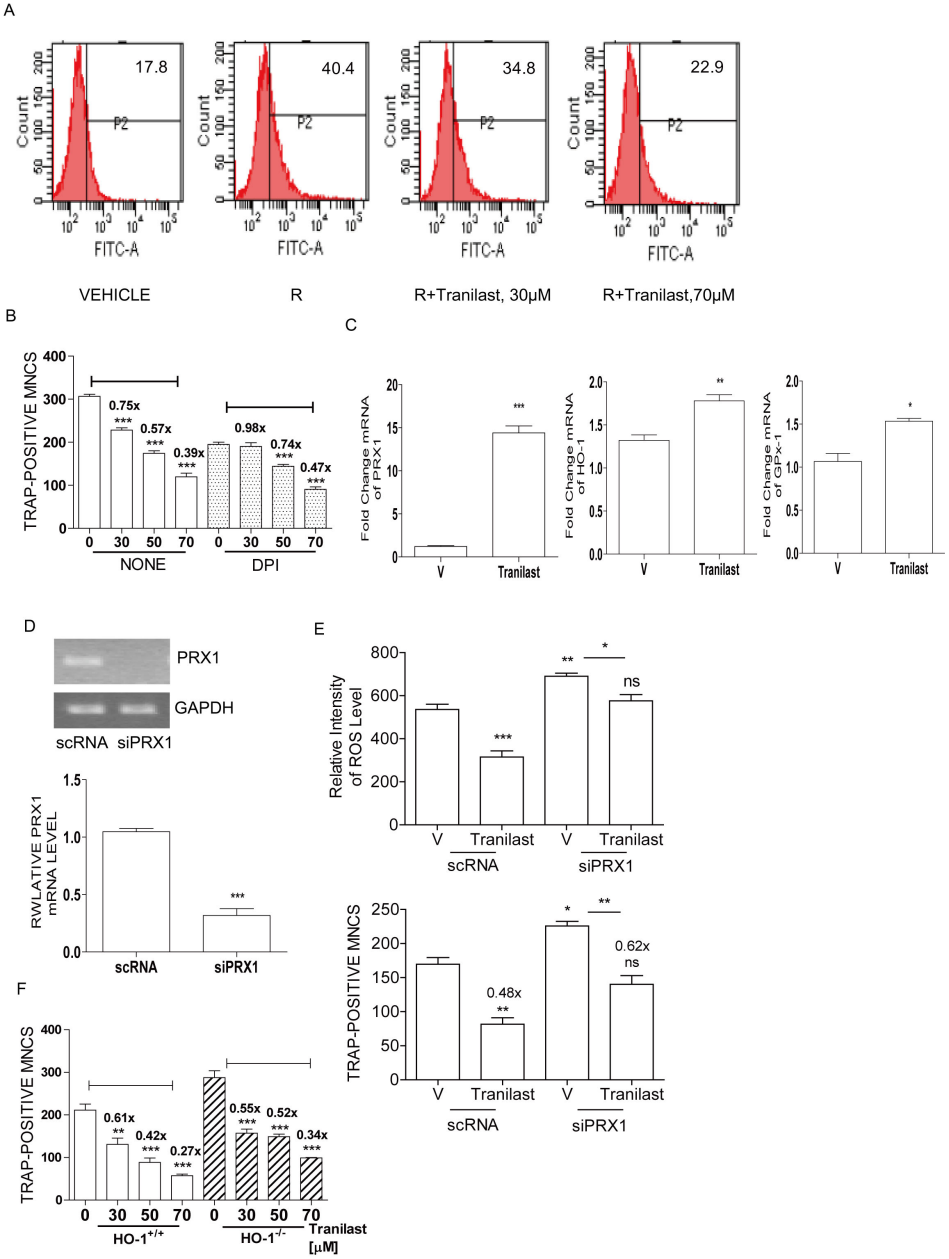
Tranilast decreases RANKL-induced ROS level. (A) Intracellular levels of ROS upon stimulation with RANKL in the presence or absence of Tranilast (30, 60 µM) for 48 h were determined using H_2_DCFDA. ROS levels were quantified by flow cytometry. (B) BMM were stimulated with M-CSF and RANKL in the presence of Tranilast (30, 50, 70 µM) with or without DPI (5 nM) for 3 d for measurement of TRAP-positive MNCs. Means of Tranilast-treated 4 groups in the presence or absence of DPI are significantly different (*P*<0.001). *** *P*<0.001 compared with each vehicle (V)-treated cells. Numbers above the histograms are ratios of the number of MNC formed in the presence of Tranilast to the number formed in its absence. (C) BMMs were incubated with 50 µM of Tranilast in the presence of M-CSF and RANKL for the indicted periods; total RNA was extracted and subjected to qPCR analysis for HO-1 (8 h), Gpx-1 or PRX1 (24 h). Expression level before RANKL treatment was set to be 1. * *P*<0.05, ** *P*<0.01, *** *P*<0.001 compared to V. (D, E) BMMs were transfected with siPRX1 or scRNA. Down-regulation of PRX1 by siRNA was confirmed by RT-PCR and qPCR. The expression levels obtained from scRNA-treated cells were set to be 1. ***, *P*<0.001 compared to scRNA (D). After 24 h of transfection with siRNA cells were treated with Tranilast and stimulated with RANKL for 48 h for determination of ROS level or 72 h for counting TRAP-positive MNCs. * *P*<0.05, ** *P*<0.01 compared with V of scRNA-transfected cells. There was a significant difference between siPRX1-treanfected V and Tranilast (* *P*<0.05; ROS, ** *P*<0.01; TRAP-positive MNC), whereas no significant difference between scRNA-transfected V and siPRX1-transfected Tranilast (ROS, TRAP-positive MNC). (E). (F) BMM from HO-1^+/+^ and HO-1^−/−^ mice were incubated in the presence of M-CSF and RANKL with Tranilast (30, 50, 70 µM). After 3 d, cells were fixed and stained for TRAP. Means of Tranilast-treated 4 groups in the presence or absence of HO-1 are significantly different (*P*<0.001). ** *P*<0.01, ***, *P*<0.001 compared with V-treated cells. Numbers above the histograms are ratios of the number of MNC formed in the presence of Tranilast to the number formed in its absence. Similar results were obtained in three independent experiments.

Since administration of Tranilast attenuated up-regulated ROS due to OVX in vivo and RANKL signaling is strongly associated with long lasting level of ROS during OC formation [15], we wondered whether Tranilast affects RANKL-induced long-lasting ROS level. ROS stimulated by RANKL was maximal at 48 h exposure (data not shown). Tranilast reduced RANKL-induced sustained level of ROS in a dose-dependent manner (**Fig. 4A**). To investigate a mechanism of decreasing ROS by Tranilast, we evaluated whether Tranilast affects ROS generation by inhibiting NADPH oxidase. Diphenylene iodonium (DPI), a selective inhibitor of NADPH oxidase reduced RANKL-induced OC formation and DPI abolished the inhibitory effect of Tranilast at 30 µM, but not completely at 50–70 µM (**Fig. 4B**), indicating another mechanism for Tranilast to inhibit OC formation. Then we searched for up-regulation of antioxidants by Tranilast in OC. Tranilast significantly increased the expression levels of peroxiredoxin 1 (PRX1), HO-1, and glutathione peroxidase 1 (Gpx-1) (**Fig. 4C**), but not thioredoxin 1 (data not shown). Knockdown of PRX1 by siRNA was confirmed by RT-PCR and qPCR (**Fig. 4D**). Down-regulation of PRX1 increased ROS level as well as OC formation upon stimulation of RANKL. It attenuated the inhibitory effect of Tranilast on ROS level and on OC formation, but not completely (**Fig. 4E**). The contribution of HO-1 to the inhibitory effect of Tranilast on OC formation was evaluated using HO-1 deficient cells. The modest decrease in inhibitory effect of Tranilast at 50–70 µM on osteoclastogenesis was observed in the absence of HO-1 (**Fig. 4F**), suggesting a partial contribution of antioxidants for action mechanisms of Tranilast.

## Discussion

The present study has demonstrated that Tranilast attenuated OVX-induced bone loss and oxidative stress in vivo. Our studies also supported the recent findings that Tranilast has other therapeutic effects in addition to being anti-allergic [12], [13], [16], [17]. The ovariectomized mice have been generally used as a model for postmenopausal bone loss. OVX-induced bone loss has been analyzed after 4 weeks of surgery using 5–6-weeks old mice [18], 8-weeks old mice [19], and 16-weeks old mice [20] with a small magnitude of difference in decreased bone density. However, OVX induced bone formation can be blunted by an immediate drop after surgery [21], but afterwards it was followed by a sustained increase [22]. Two weeks after OVX result in a trend of decrease of perimeters of osteoblast [21], whereas 4 weeks after surgery increased serum osteocalcin [22]. Our model has been analyzed after 8 weeks of surgery using 6-weeks old mice with increased bone formation due to OVX. Tranilast reduced the increase of serum CTX-1 and ex vivo cultured OC upon OVX, suggesting that Tranilast protects from bone loss via acting in OC. Consistently RANKL-induced osteoclastogenesis was decreased by Tranilast in vitro. The inhibitory effect of Tranilast was caused by the delay of commitment to OC, since neither OC survival nor bone resorption was affected by Tranilast. It is not likely that bone-sparing effects observed could be due to stimulation of bone formation, since serum ALP and osteocalcin, in vivo bone formation marker [23], were not affected by administration of Tranilast. It was also supported by the finding that ex vivo OCs from whole bone marrow showed a similar tendency as those from BMM, excluding the contribution of stromal/osteoblast cells to the protective effect of Tranilast.

Among two essential signals for osteoclastogenesis, we focused RANKL signaling to investigate the inhibitory effect of Tranilast on OC formation, since Tranilast did not affect proliferation of BMM upon M-CSF stimulation. Our data demonstrated that Tranilast decreased RANKL-induced NF-κB DNA binding activity dose-dependently. Importance of NF-κB in bone metabolism has been demonstrated by p50/p52 double knockout mice, developing typical osteopetrosis with a primary defect with OC lineage cells [24]. The inhibitory effect of Tranilast on NF-κB activation has been demonstrated in other cells. Tranilast inhibits MCP-1 secretion in rat mesengial cells [25] and iNOS expression and activity in microglial cells [26] by inhibiting NF-κB activity. Tranilast also decreases NF-κB-dependent transcriptional activation in endothelial cells [27]. NFAT2 has been identified to be the prominent target gene induced by RANKL during osteoclastogenesis [6]. Our data showed that Tranilast decreased the expression of NFAT2 at mRNA and protein level as well as nuclear translocation upon RANKL stimulation, suggesting a decreased NFAT2 activity in OC by Tranilast. The reduced expression of c-Fos also supported an impaired NFAT2 activity. RANKL stimulation induces to bind to DNA through ternary complex formation with c-Fos. Deficiency of c-Fos results in failure of induction of NFAT2 on RANKL stimulus with no OC [28], suggesting a critical role of c-Fos in activation of NFAT2 and OC formation. Since amplification and activation of NFAT2 are associated with a long lasting level of ROS via Ca^2+^ oscillation [15], decreased NFAT2 activity could be due to reduced ROS induced by Tranilast. Alternatively, it could be partly due to attenuated RANKL-induced TGF-β expression by Tranilast, since exogenous TGF-β recovered partially attenuated mRNA level of NFAT2 and OC formation by Tranilast.

RANKL-induced ROS generation is essential for osteoclastogenesis [15], [29]. Tranilast reduced substantial amount of sustaining level of ROS induced by RANKL. Consistently elevated serum ROS upon OVX was decreased by Tranilast in vivo, indicating that administration of Tranilast reduces oxidative stress induced by loss of ovarian function. Decreased ROS level by Tranilast can be explained by its abilities not only to inhibit ROS generation but also to induce antioxidant enzymes such as HO-1, PRX1, and Gpx-1 in OC. The role of Tranilast to decrease ROS also has been demonstrated in other studies. Tranilast reduces ROS generated from zymogen-stimulated polymorphonuclear leukocytes [30], and also decreases oxidative stress in diabetic nephropathy by modulating thioredoxin [17].

Taken together, our results have demonstrated that Tranilast protected against OVX-induced bone loss in mice. Tranilast inhibited OC formation via impaired RANKL signaling with decreased activations of NF-κB and NFAT2, and reduced levels of ROS as well as TGF-β. These results suggested that Tranilast may be beneficial in alleviating bone loss as well as an oxidative stress caused by loss of ovarian function. It is possible that Tranilast could be a potent candidate to reduced post-menopausal bone loss beyond the present usage of an anti-allergic drug, although it requires further studies.

## Materials and Methods

### Ethics Statement

All mice were handled in accordance with the guidelines of the Institutional Animal Care and Use Committee (IACUC) of the Immunomodulation Research Center (IRC), University of Ulsan. All animal procedures were approved by IACUC of IRC. The approval ID for this study is #2011-001.

### Animals and Study Design

Six-week-old C57BL/6J mice were subjected to sham operation (n = 12), or ovariectomy (OVX) (n = 12) under anesthesia using mixture of Zoletil and Rompun. All mice were housed in the specific pathogen-free animal facility of IRC. Tranilast (generously provided by KISSEI Pharmaceutical Co., Ltd, Nagoya, Japan) was suspended in 0.5% methylcellulose solution and administered intragastrically at dose of 200 mg/kg (OVX, n = 6; sham, n = 6) or vehicle (OVX, n = 6; sham, n = 6), once a day for 8 weeks after surgery. The mice were sacrificed by CO_2_ asphyxiation. For visualization and architecture of long bone, femurs were scanned in a high-resolution Micro CT (µCT) imaging system using the SkyScan 1072 System (Sky Scan) with setting to a 6.9 µm effective detector pixel size and a threshold of 77–255 mg/cc. Trabecular bone was analyzed in the 1.5 mm region in length, 0.2 mm below the distal growth plate of femurs. Total of 250–300 tomographic slices were acquired and 3 D analyses were performed with CT volume software (ver 1.11; SkyScan). In vivo markers of bone resorption and OC numbers were measured according to the manufacturer’s directions (Immunodiagnostic Systems Inc., Woburn, MA): serum collagen-type I fragments (CTX-1) by RatLaps EIA and serum TRACP5b by solid phase immune-fixed enzyme activity assay, respectively. Serum osteocalcin was determined by osteocalcin EIA kit (Biomedical Technologies Inc., USA) and alkaline phosphatase (ALP) by a colorimetric kinetic determination (BioAssay Systems, Hayward, CA, USA) respectively. Serum H_2_O_2_ was determined by Amplex Red hydrogen peroxide/peroxidase assay kit (Invitrogen, Carlsbad, CA).

### OC Formation

Bone marrow cells were isolated from 4–5-week-old C57BL/6J mice as described before [31]. HO-1^−/−^ mice in the background of Balb/c were generously provided by Dr. Perrella MA (Harvard Medical School). Femora and tibiae were removed aseptically and dissected free of adherent soft tissue. The bone ends were cut, and the marrow cavity was flushed out with α-MEM from one end of the bone using a sterile 21-gauge needle for further agitation using a Pasteur pipette to get a single cell suspension. The resulting bone marrow suspension was washed twice, and incubated on plates along with M-CSF (20 ng/ml) for 16 h. Non-adherent cells were then harvested, layered on a Ficoll-hypaque gradient for collecting cells at the interface, and cultured for two more days, at which time large populations of adherent monocyte/macrophage-like cells had formed on the bottom of the culture plates as described before [31]. The small numbers of non-adherent cells were removed by washing the dishes with phosphate-buffered saline (PBS), and the remaining adherent cells (bone marrow-derived macrophages (BMM)) were harvested, and seeded in plates. The adherent cells were analyzed by a FACSCalibur flow cytometer (Becton Dickinson, Franklin Lakes, NJ) and found to be negative for CD3 and CD45R, and positive for CD11b. Absence of contaminating stromal cells was confirmed by lack of growth without addition of M-CSF. Additional medium containing M-CSF and RANKL (40 ng/ml) was added, and the medium was replaced on day 3. After incubation for the indicated times, the cells were fixed in 10% formalin for 10 min, and stained for tartrate-resistant acid phosphatase (TRAP) as described [31]. Numbers of TRAP-positive multinucleated cells (MNC) (three or more nuclei) were scored. BMM was transfected with small interfering RNA (siRNA) against peroxiredoxin 1 (siPRX1) or scrambled siRNA (scRNA) (Santa Cruz, Santa Cruz, CA) using Lipofectamine™ RNAiMAX (Invitrogen). Lipofectamine RNAiMAX (Invitrogen) was first diluted in a-MEM and mixed with an equal volume of a-MEM containing the siRNA. After 20 min of incubation, 100 µl of the resulting RNAiMAX/siRNA was added directly onto the cells, giving a final volume of 700 µl. After 8h incubation, the cells were replated in serum-containing medium and cultured for another 2 d. mRNA expression was analyzed by qPCR. OC were further characterized by assessing their ability to form pits on dentine slices, as described [32]. Mature OC cells were generated by incubation with M-CSF and RANKL for 5 d. Then, after treatment with EDTA, the cells were harvested [33]. Mature OCs were seeded on dentine slices and incubated for 1 d with M-CSF and RANKL. The slices were cleaned by ultrasonication in 1M NH_4_OH to remove adherent cells and stained with Mayer’s hematoxylin (Sigma) to visualize resorption pits.

### RNA Isolation and Quantitative Polymerase Chain Reaction (qPCR)

Total RNA was reverse-transcribed with oligo-dT and Superscript I (Invitrogen, Carlsbad, CA). qPCR was carried out using SYBR Green 1 Taq polymerase (Qiagen, Hilden, Germany) and appropriate primers on a DNA Engine Opticon Continuous Fluorescence Detection System (MJ Research Inc., Waltham, MA). The specificity of each primer pair was confirmed by melting curve analysis and agarose-gel electrophoresis. The housekeeping GAPDH gene was amplified in parallel with the genes of interest. Relative copy numbers compared to GAPDH were calculated using 2^−ΔΔCt^. The primer sequences used were as follows: 5′-ctgctcctagtgagcccaac-3′ and 5′-cagcaatcgacaaggagtga-3′ (calcitonin receptor); 5′-gaccaccttggcaatgtctctg-3′ and 5′-tggctgaggaagtcatctgagttg-3′ (TRAP); 5′-agcctttcctactaccattccc-3′ and 5′-tggcactagagacggacaga-3′ (c-Fos); 5′-aataacatgcgagccatcatc-3′ and 5′-tcaccctggtgttcttcctc-3′ (NFAT2); 5′-gctcccctatttaagaacacccac-3′ and 5′-ctcccaaggaaaggtaggtgatag-3′ (TGF-β); 5′-gggactacaccgagatgaacga-3′ and 5′-accattcacttcgcacttctca-3′ (Gpx-1); 5′-tcccagacaccgctcctccag-3′ and 5′-ggatttggggctgctttc-3′ (HO-1); 5′-tgccagatggacaattcaaa-3′ and 5′-cagctggacacacttcacca-3′ (PRX1); 5′-acccagaagactgtggatgg-3′ and 5′-cacattgggggtaggaacac-3′ (GAPDH).

### EMSA

Biotinylated double-stranded oligonucleotides were synthesized by Bioneer Co. (Korea): NF-κB, 5′-agttgaggggactttcccaggc-3′; NF-Y, 5′-agaccgtacgtgattggttaatctctt- 3′. Nuclear extracts were prepared from BMM cells stimulated with RANKL (40 ng/ml) using NE-PER nuclear and cytoplasmic extraction reagents (Pierce) according to the manufacturer’s manual. Binding reactions were carried out for 20 min at room temperature in the presence of 50 ng/ml poly(dI-dC), 0.05% Nonidet P-40, 5 mM MgCl_2_, 10 mM EDTA, and 2.5% glycerol in 1× binding buffer using 20 fM of biotin-end-labeled target DNA and 3 µg of nuclear extract according to the manufacturer’s manual (LightShift^TM^Chemiluminescent EMSA kit; Pierce). Samples were loaded onto native 6% polyacrylamide gels pre-electrophoresed for 60 min in 0.5x Tris borate/EDTA and electrophoresed at 100 V before being transferred onto a positively charged nylon membrane (Hybond™-N+) in 0.5 X Tris borate/EDTA at 100 V for 30 min. Transferred DNAs were cross-linked to the membrane at 10 mJ/cm^2^ and detected using horse radish peroxidase (HRP)-conjugated streptavidin.

### Fractionation and Western Blot Analysis

Cultured cells were harvested after washing with ice-cold PBS and then lysed in extraction buffer (50 mM Trsi-HCl, pH 8.0, 150 mM NaCl, 1 mM EDTA, 0.5% Nonidet P-40, 0.01% protease inhibitor mixture). Cells were fractionated using Nuclear and Cytoplsmic Extraction reagents (Pierce) according to the manufacturer’s protocol. Cytoplasmic and nuclear extracts were subjected to SDS-PAGE and transferred onto nitrocellulose. Membranes were blocked for 1 h with skim milk in Tris-buffered saline containing 0.1% Tween 20% and incubated overnight at 4°C with anti-NFAT2 (0.2 µg/ml), anti-Lamin B1 (0.1 µg/ml; Santa Cruz) and anti-β-actin (2.2 µg/ml; Sigma). Membranes were washed, incubated for 1 h with HRP-conjugated secondary Abs (BD Biosciences), and developed using chemiluminescence substrates.

### Intracellular Reactive Oxygen Species (ROS) Detection

The intracellular formation of ROS was detected using the fluorescence probe 2′,7′-dichlorofluorescein diacetate (H_2_DCFDA) (Molecular Probes). After BMMs were cultured under the different experimental conditions for 48 h, the cells were harvested, suspended in PBS, loaded with H_2_DCFDA, and incubated at 37°C for 30 min. The measurement of intracellular ROS was performed using flow cytometry with a FACSCalibur.

### Statistical Analysis

Values are expressed as means ± SEM. Statistical analysis was performed by Student’s *t*-test when two groups were compared or by one-way ANOVA, followed by Bonferroni posttests if multiple groups were compared. A *P* value of less than 0.05 was considered statistically significant.

## References

[1] Manolagas SC (2000) Birth and death of bone cells: basic regulatory mechanisms and implications for the pathogenesis and treatment of osteoporosis. Endocr Rev 21: 115–137. 10782361 10.1210/edrv.21.2.0395

[2] Weitzmann M, Pacifici R (2006) Estrogen deficiency and bone loss: an inflammatory tale. J Clin Invest 116: 1186–1194. 16670759 10.1172/JCI28550 PMC1451218

[3] Roodman GD (1996) Advances in bone biology: the osteoclast. Endocrinol Rev 17: 308–332. 10.1210/edrv-17-4-308 8854048

[4] Suda T, Takahashi N, Udagawa N, Jimi E, Gillespie MT, et al (1999) Modulation of osteoclast differentiation and function by the new members of the tumor necrosis factor receptor and ligand families. Endocrinol Rev 20: 345–357. 10.1210/edrv.20.3.0367 10368775

[5] Kong YY, Yoshida H, Sarosi I, Tan HL, Timms E, et al. (!999) OPGL is a key regulator of osteoclastogenesis, lymphocyte development and lymph-node organogenesis. Nature 397: 315–323. 9950424 10.1038/16852

[6] Takayanagi H, Kim S, Koga T, Nishina H, Isshiki M, et al (2002) Induction and activation of the transcription factor NFATc1 (NFAT2) integrate RANKL signaling in terminal differentiation of osteoclasts. Dev Cell 3: 889–901. 12479813 10.1016/s1534-5807(02)00369-6

[7] Hirotani H, Tuohy NA, Woo JT, Stern PH, Clipstone NA (2004) The calcineurin/nuclear factor of activated T cells signaling pathway regulates osteoclastogenesis in RAW264.7 cells. J Biol Chem 279: 13984–13992. 14722106 10.1074/jbc.M213067200

[8] Matsumoto M, Kogawa M, Wada S, Takayanagi H, Tsujimoto M, et al (2004) Essential role of p38 mitogen-activated protein kinase in cathepsin K gene expression during osteoclastogenesis through association of NFATc1 and PU.1. J Biol Chem 279: 45969–45979. 15304486 10.1074/jbc.M408795200

[9] Komatsu H, Kojima M, Tsutsumi N, Hamano S, Kusama H, et al (1988) Study of the mechanism of inhibitory action of tranilast on chemical mediator release. Jpn J Pharmacol 46: 43–51. 2452912 10.1254/jjp.46.43

[10] Platten M, Ho PP, Youseff S, Fontoura P, Garren H, et al.. (2005) Treatment of autoimmune neuroinflammation with a synthetic tryptophan metabolite. Science 310; 850–855. 10.1126/science.1117634 16272121

[11] Pae HO, Jeong SO, Koo BS, Ha HY, Lee KM, et al (2008) Tranilast, an orally active anti-allergic drug, up-regulates the anti-inflammatory heme oxygenase-1 expression but down regulates the proinflammatory cyclooxygenase-2 and inducible nitric oxide synthase expression in RAW 264.7 macrphages. Biochem Biophys Res Commun 371: 361–365. 18435907 10.1016/j.bbrc.2008.04.054

[12] Sato S, Takahashi S, Asamoto M, Naiki T, Naiki-Ito A, et al (2010) Tranilast suppresses prostste cancer growth and osteoclast differentiation in vivo and in vitro. The Prostate 70: 229–238. 19790239 10.1002/pros.21056

[13] Uno M, Kurita S, Misu H, Ando H, Ota T, et al (2008) Tranilast, an antifibrogenic agent, ameliorates a dietary rat model of nonalcoholic steatohepatitis. Hepatology 48: 109–118. 18571789 10.1002/hep.22338

[14] Fox SW, Evans KE, Lovibond AC (2008) Transforming growth factor-beta enables NFATc1 expression during osteoclastogenesis. Biochem Biophys Res Commun 366: 123–128. 18060870 10.1016/j.bbrc.2007.11.120 PMC2568814

[15] Kim MS, Yang Y, Son A, Tian YS, Lee SI, et al (2010) RANKL-mediated reactive oxygen species pathway that induces long lasting Ca^2+^ oscillations essential for osteoclastogenesis. J Biol Chem 285: 6913–6921. 20048168 10.1074/jbc.M109.051557 PMC2844141

[16] Shiota N, Kovanen PT, Eklund KK, Shibata N, Shimoura K, et al (2010) The anti-allergic compound tranilast attenuates inflammation and inhibits bone destruction in collagen-induced arthritis in mice. Br J Pharmacol 159: 626–635. 20067475 10.1111/j.1476-5381.2009.00561.x PMC2828026

[17] Tan SM, Zhang Y, Cox AJ, Kelly DJ, Qi W (2011) Tranilast attenuates the upregulation of thioredoxin-interacting protein and oxidative stress in an experimental model of diabetic nephropathy. Nephrol Dial Transplant 26: 100–110. 20573806 10.1093/ndt/gfq355

[18] Lee SK, Kadono Y, Okada F, Jacquin C, Koczon-Jaremko B, et al (2006) T lymphocyte-deficient mice lose trabecular bone mass with ovariectomy. J Bone Miner Res 21: 1704–1712. 17002560 10.1359/jbmr.060726

[19] Lee SK, Kalinowski JF, Jacquin C, Adams DJ, Gronowicz G, et al (2006) Interleukin-7 influences osteoclast function in vivo but is not a critical factor in ovariectomy-induced bone loss. J Bone Miner Res 21: 695–702. 16734384 10.1359/jbmr.060117

[20] Gao Y, Grassi F, Ryan MR, Terauchi M, Page K, et al (2007) IFN-gamma stimulates osteoclast formation and bone loss in vivo via antigen-driven T cell activation. J Clin Invest 117: 122–132. 17173138 10.1172/JCI30074 PMC1697800

[21] Li JY, Tawfeek H, Bedi B, Yang X, Adams J, et al (2011) Ovariectomy disregulates osteoblast and osteoclast formation through the T-cell receptor CD40 ligand. Proc Natl Acad Sci U S A 108: 768–773. 21187391 10.1073/pnas.1013492108 PMC3021053

[22] Cenci S, Weitzmann MN, Roggia C, Namba N, Novack D, et al (2000) Estrogen deficiency induces bone loss by enhancing T-cell production of TNF-alpha. J Clin Invest 106: 1229–1237. 11086024 10.1172/JCI11066 PMC381439

[23] Kim HJ, Zhao H, Kitaura H, Bhattacharyya S, Brewer JA, et al (2006) Glucocorticoids suppress bone formation via the osteoclast. J Clin Invest 116: 2152–2160. 16878176 10.1172/JCI28084 PMC1518793

[24] Iotsova V, Okan I, Fritsche M, Strom M, Groner B, et al (1997) Osteopetrosis in mice lacking NF-κB1 and NF-κB2. Nat Med 3: 632–638. 9176489 10.1038/nm0697-632

[25] Chikaraishi A, Hirahashi J, Takase O, Marumo T, Hishikawa K, et al (2001) Tranilast inhibits interleukin-1beta-induced monocyte chemoattractant protein-1 expression in rat mesangial cells. Eur J Pharmacol 427: 151–158. 11557268 10.1016/s0014-2999(01)01215-8

[26] Platten M, Wick W, Wischhusen J, Weller M (2001) N-[3,4-dimethoxycinnamoyl]-anthranilic acid (tranilast) suppresses microglial inducible nitric oxide synthase (iNOS) expression and activity induced by interferon-gamma (IFN-gamma). Br J Pharmacol 134: 1279–1284. 11704648 10.1038/sj.bjp.0704373 PMC1573061

[27] Spiecker M, Lorenz I, Marx N, Darius H (2002) Tranilast inhibits cytokine-induced nuclear kappaB activation in vascular endothelial cells. Mol Pharmacol 62: 856–863. 12237332 10.1124/mol.62.4.856

[28] Wagner EF, Eferi R (2005) Fos/AP-1 proteins in bone and the immune system. Immunol Rev 208: 126–140. 16313345 10.1111/j.0105-2896.2005.00332.x

[29] Lee NK, Choi YG, Baik JY, Han SY, Jeong D, et al (2005) A crucial role for reactive oxygen species in RANKL-induced osteoclast differentiation. Blood 105: 852–859. 10.1182/blood-2004-09-3662 15817678

[30] Miyachi Y, Imamura S, Niwa Y (1987) The effect of Tranilast of generation of reactive oxygen species. J Pharmacobiodyn 10: 255–259. 2444694 10.1248/bpb1978.10.255

[31] Lee JE, Shin HH, Lee EA, Phan TV, Choi HS (2007) Stimulation of osteoclastogenesis by enhanced level of MIP-1alpha in BALB/c mice. Exp Hematol 35: 1100–1108. 17588479 10.1016/j.exphem.2007.04.006

[32] Jimi E, Akiyama S, Tsurukai T, Okahashi N, Kobayashi K, et al (1999) Osteoclast differentiation factor acts as a multifunctional regulator in murine osteoclast differentiation and function. J Immunol 163: 434–442. 10384146

[33] Fuller K, Kirstein B, Chambers TJ (2006) Murine osteoclast formation and function: differential regulation by humoral agents. Endocrinology 147: 1979–1985. 16384864 10.1210/en.2005-1340

